# The Sweating Sicknesses

**Published:** 1921-11

**Authors:** A. T. Nankivell

**Affiliations:** (Medical Officer of Health, Hornsey)


### NOTABLE EPIDEMICS.

### II. THE SWEATING SICKNESSES.

By A. T. Nankivell, M.D., D.P.H. (Medical Officer of Health, Hornsey).

In the year 1485 Henry Tudor landed at Milford Haven with an army of foreign mercenaries, and, marching to Bosworth Field, defeated Richard III. on August 22. He made his triumphant entry into London on August 27, and brought with him in his train "one by no means a captive." A deadly sickness broke out in the capital on September 19, so that more than fifteen thousand persons were cut off by sudden death, as if by the visitation of God, many dying while walking in the streets without warning. There was a certain Dr. Thomas Forrestier, who lived and wrote in those days, and he has left us a record of this disease—a record meagre enough in scientific detail, but nevertheless a human document. The Sweating Sickness, or, as it was called, the "English Sweat," was probably our first experience of influenza; and the occurrence of this new disease upon a new soil resulted in a very severe type of the disease associated with high mortality. An attack of the Sweat came on with explosive suddenness, and death was swift. This is what Dr. Forrestier writes about it:

"We saw two priests standing together and speaking together, and we saw both of them die suddenly. Also on the next day we see the wife of a tailor taken and suddenly died. Another young man walking by the street fell down suddenly. Also another gentleman riding out of the City died." High and low, rich and poor, one with another fell suddenly to the new disease. Two mayors, four aldermen, and many worshipful commoners perished in the City. The Sweat, which began in September, "continued till the end of October, of the which a wonderful number died."

The signs and symptoms of the Sweat are given as follows: "This sickness cometh with a great sweating and stinking, with redness of the face and of all the body, and a continual thirst, with a great heat and headache because of the fumes and venoms. . . . Some appear red and yellow, as we have seen many, and in two great ladies that we saw, the which were sick in all their bodies, and they felt great pricking in their bodies, and some had black spots . . . " These latter were probably hæmorrhagic cases, such as proved so rapidly fatal in our last influenza epidemic.

The first English Sweat lasted for about six weeks in London, and no doubt spread from there throughout the country, for we hear of it at Oxford, where it "lasted but a month or six weeks," and killed many of the scholars before they could go to their homes. After this nothing more is heard of the English Sweat for twenty-three years, when it reappeared in 1508, again taking a heavy toll of life. The disease came again in 1517, yet again in 1528, and for the last time in 1551. Thus in seventy years or less there were five great outbreaks in England of this rapidly fatal disease.

With each successive epidemic, as is natural, our knowledge of the disease becomes more plentiful; and to illustrate the fifth and last English Sweat of 1551 we have two writings by Dr. Caius, who was the Court physician to Henry VIII. and also to Edward VI. The last great epidemic of Sweat began apparently in April at Shrewsbury, where it killed some 900 persons, and passed slowly to London, arriving at the beginning of July. It was epidemic in London about a month. People found new names for their fifth visitation of the epidemic, and it was known as "the Sweat called New Acquaintance, alias Stop-Knave-and-Know-thy Master." It appears also in parish registers as "Stop-gallant"; and we read "God had plagued this realm most justly with . . . the Posting Sweat, that posted from town to town throughout England, and was named Stop-gallant, for it spared none: for there were some dancing in the Court at nine o'clock that were dead at eleven." We read also that all business was suspended in London, the shops were closed, and that none had care for anything save for the preservation of life, and that during the first three days of the Sweat in London there were five thousand deaths. Probably this estimate of the London mortality is exaggerated; but, at any rate, the Sweat, which killed men without warning and without their being confessed, paralysed the life of the City as much as did the visitation of the Plague.

### Epidemiology.

In the year 1529 the English Sweat appeared on the Continent of Europe, and spread through all the countries with the notable exception of France, Spain, and Italy. With regard to Italy and Spain, shut off as they are by the Alps and Pyrenees from land communication with the rest of Europe, the explanation of their immunity is not difficult. The slow journey across the mountain stopped not only the gallants but also the Stop-gallant. The immunity of France is not so easy to explain, unless we assume that the Sweat was a disease endemic and therefore mild in type in France, and that the population there was relatively immune. Granted this and remembering that Henry VII. brought with him French mercenaries to win the battle of Bosworth, we are given a reasonable explanation both of the introduction of influenza, or the Sweat, into England by the French troops, and also of the immunity of France to the disease. It was not until the year 1717 that a "Sweat" like the English Sweat broke out in Picardy, and it is reasonable to suppose that between 1529 and 1717 the French nation may have lost some of their natural immunity. We will consider shortly for the sake of elaborating this argument what might happen on half-a-dozen islands in the South Seas if measles were an endemic disease on one of the islands and

**Notable Epidemics—(*cont.*).**

had never been known upon any of the others. One day an expedition sets out from the measles island and reaches one of the other islands: in that latter place measles becomes established. There is a series of epidemics characterised by high mortality. After the fourth epidemic the inhabitants scatter, and go on visits to the other islands in the group, and they introduce measles into all the other islands with the exception of that on which measles had been endemic for so long. A couple of hundred years or so go by and measles is re-introduced into the island where once it was endemic, but owing to the lapse of two centuries the immunity of the people has declined and the disease gains a foothold once more. This is not altogether an exaggerated picture of the epidemiology of the English Sweats, of their introduction into England from an endemic focus, and of their spread over the Continent of Europe.

### Disappearance or Modification?

The Sweats in England caused a vast mortality during their five visitations between 1485 and 1551, but what happened to them then? We have no more records of English Sweats or of mortality from them until the influenza epidemics of the last hundred years or so, and even these influenza epidemics were mild and trifling afflictions when compared to the original English Sweats. Did the disease really disappear like leprosy, or did it become modified in form and less virulent in type? Probably the latter is the explanation of the apparent departure of the Sweat. On introduction into England it was the new disease on the new soil, and ran through the country like fire among the dried grass; after its fifth appearance it became modified in type, and the English people began to acquire some immunity to the disease. That fevers were common from the sixteenth century down to our own times we know well, but they were not accompanied by any marked mortality, as were the Sweats. Influenzas, hot agues, universal colds, malignant fevers, and transient fevers became known to the people. They still suffered from the Sweat, but in a milder form; and because it did not kill them as they walked the streets, but merely laid them up for a few days at home, they found other and less terrible names for their enemy. We can explain in the same manner the apparent departure of influenza from this country between 1891 and 1918. The disease was epidemic and fatal at these two periods; but during the intervening time it gave rise to a small mortality. During the months of the epidemic all catarrhal, febrile, and bronchial affections tended to be spoken of as influenza; but in non-epidemic times the people suffered from colds in the head, a chill, a chill on the liver, a touch of bronchitis, or a cold on the chest. It is likely that many of these non-fatal catarrhal conditions are really due to the virus of influenza; but as they are transient and non-fatal they are not labelled with the name of the terrible epidemic disease.

### GOVERNMENT OF GREATER LONDON.

**Members of the Royal Commission.**

It is announced that the King has approved the following terms of reference for the Royal Commission on the Local Government of Greater London:

To inquire and report what, if any, alterations are needed in the local government of the administrative County of London and the surrounding districts, with a view to securing greater efficiency and economy in the administration of local government services and to reducing any inequalities which may exist in the distribution of local burdens as between different parts of the whole area.

The members of the Commission are as follow: The Right Hon. the Viscount Ullswater, G.C.B. (chairman), Sir Richard Vassar-Smith, Bt.; Sir Albert Gray, K.C., K.C.B., Sir Horace Cecil Monro, K.C.B., Mr. George John Talbot, K.C., Mr. Neville Chamberlain, M.P., Mr. Robert Donald, LL.D., Mr. Ernest Haviland Hiley, C.B.E., Mr. Edmund Russborough Turton, M.P. There will also be a representative of Labour, whose name will be subsequently announced.

The secretary of the Commission is Mr. Michael Heseltine, C.B., of the Ministry of Health, Whitehall, S.W. 1, to whom all communications relating to the work of the Commission should be addressed.

### RECIPROCAL REGISTRATION.

Now that the Scottish Registration Rules have been accepted as framed and the vexed question of the admission of the Scottish Board of Health's Fever Nurses to the Register is settled, nothing should stand in the way of arranging reciprocity between the three parts of the kingdom. The Scottish Board of Health and the Secretary for Scotland fully appreciate that reciprocity of Registration is the ruling consideration in the interests of the whole body of nurses in Scotland. Sir Alfred Mond has brought the point before the English General Nursing Council, which has approved a rule already drafted, providing for reciprocal Registration, with a uniform standard of qualification, as between all parts of the United Kingdom. The English Council has, however, decided not to reduce its fee in the case of intermediate nurses coming on to its Register under reciprocal Registration, which will remain the same as for the original Registration, i.e. two guineas. Under the Scottish Rules the fee for intermediate nurses is one guinea, while the Irish Rules provide for a similar fee until January 1, 1922, when it will be raised to two guineas.

Messrs. E. & S. Livingstone, the well-known medical and scientific publishers, of Edinburgh, have undertaken the British and Colonial Agency of "The American Medical Record." This weekly journal of medicine and surgery is published in New York, and is fifty-five years old.

